# The Influence of Microfungi on the Mycelial Growth of Ectomycorrhizal Fungus *Tricholoma matsutake*

**DOI:** 10.3390/microorganisms7060169

**Published:** 2019-06-07

**Authors:** Seung-Yoon Oh, Myung Soo Park, Young Woon Lim

**Affiliations:** School of Biological Sciences and Institute of Microbiology, College of Natural Sciences, Seoul National University, Seoul 08826, Korea; syoh@snu.ac.kr (S.-Y.O.); ms1014@snu.ac.kr (M.S.P.)

**Keywords:** fungal metabolite, growth promoting fungus, *Mortierella*, *Penicillium*, pine mushroom, *Trichoderma*, *Umbelopsis*, zygomycota

## Abstract

Pine mushroom (*Tricholoma matsutake*) is one of the most valued ectomycorrhizal fungi in Asia because of its unique pine-like aroma; however, due to exceptionally slow growth of its mycelia in artificial conditions, its cultivation has been largely deemed as not possible. Previous studies have shown that some bacteria and a few *Trichoderma* species associated with pine mushroom promoted the growth of *T. matsutake* isolate, but this effect is relatively unexplored. In this study, we investigated the diversity of microfungi in the fairy ring of *T. matsutake* and their effect on the growth of *T. matsutake* isolate. From 184 fungal isolates, 28 species were identified based on suitable molecular markers. *Penicillium* was most frequently observed (16 species), followed by *Trichoderma* (4 species). Five Zygomycota species showed a high promoting effect on the growth of *T. matsutake* while the effects of ascomycetes were mixed. The microfungi that promote the growth of *T. matsutake* can be useful for forest nursery and artificial cultivation of *T. matsutake*.

## 1. Introduction

Ectomycorrhizal fungi (EMF) play a crucial role in plant development and the nutrient cycle of the forest ecosystem [1,2]. Ectomycorrhizae are specialized symbiotic structures combining fungal hyphal systems with host root systems for nutrient interchange [3]. To form ectomycorrhizae, however, the hyphae of EMF need to overcome several challenges, such as initial host recognition, host immune defense, and viability at a pre-symbiotic stage in soil [4]. Recent studies have shown that microorganisms co-existing with EMF (e.g., mycorrhiza helper bacteria (MHB)) facilitate mycorrhizal formation [5]. These microorganisms promote the mycelial growth of EMF in order to prepare the fungi for mycorrhizal colonization [5] and to increase the nutrient foraging ability of EMF, making them an attractive symbiotic partner [6,7].

Microfungi were also found in various structures of EMF such as ectomycorrhizae [8,9], hyphae [10], and fruiting bodies [11,12]. In the rhizosphere, EMF and microfungi live together, sharing or competing for resources such as nutrients or space [13,14,15]. EMF suppress the growth of saprotrophic microfungi [16] and pathogenic microfungi to protect the hosts’ health [17,18]. Reversely, microfungi also suppress ectomycorrhizal colonization, hyphal growth, and spore germination of EMF [15]. Little has been known, however, about the positive effect of microfungi on ectomycorrhizal development [19,20].

*Tricholoma matsutake* (pine mushroom (PM)) is an ectomycorrhizal fungus, and its fruiting bodies are edible and highly prized in Asia [21]. PM has a symbiotic relationship with trees belonging to Fagacea and Pinaceae, such as *Pinus densiflora* and *Quercus serrata* [22]. Around the host trees, PM forms a hyphal dominant zone known as the fairy ring owing to its arc-shaped morphology [21]. In previous studies, specific microfungi have been frequently isolated from PM fairy rings [23,24,25,26]. Recently, distinct microbial communities in PM fairy rings have been detected using a culture-independent approach [27,28,29].

In our previous studies, we showed that several bacterial species isolated from PM fairy rings are able to promote PM mycelial growth [30,31]. As for the fungal species, Ogawa and Kawai [32] reported that metabolites of *Mortierella* species promoted the hyphal growth of PM about twofold. Similarly, metabolites of some *Trichoderma* species isolated within PM fairy rings also promoted PM growth [33]. We speculated that other microfungi that promote PM mycelial growth can be found within the PM fairy rings. In this study, we isolated microfungi from soil within PM fairy rings and identified them using appropriate molecular markers, such as the nuclear ribosomal internal transcribed spacer (ITS) [34] and protein-coding genes. In addition, we extracted metabolites from PM-associated microfungi and determined their effects on the mycelial growth of PM.

## 2. Materials and Methods

### 2.1. Sample Collection and Microfungal Isolation

Soil samples were collected from three PM fairy rings at each of four sampling locations in September 2013. The four sampling locations were selected based on the reported amount of PM occurrence: Hongcheon County (N37° 41′ 49″ E127° 53′ 19″, altitude: 438 m), Uljin County (N36° 59′ 05″, E129° 06′ 09″, altitude: 167 m), Yeongdeok County (N36° 24′ 36″, E129° 21′ 24″, altitude: 372 m), and Pohang (N36° 06′ 57″, E129° 08′ 51″, altitude: 254 m). The vegetation in the sampling locations is mixed, mainly composed of *Pinus densiflora* with a small number of broadleaf trees (e.g., *Quercus* spp. and *Rhododendron* spp.). The fairy rings were found under *Pinus densiflora,* where the production of PM fruiting bodies has been monitored annually. Each fairy ring within the same location was more than 50 m away. The active PM hyphal area in the fairy ring was chosen as a sampling point based on soil color (greyish white) and hyphal structure by eye. After removing the litters, we collected a single soil block (10 cm × 5 cm) at a depth of 0–5 cm using a sterilized spatula. About 50 g of soil samples were collected and transferred to the laboratory in the icebox. For microfungal isolation, potato dextrose agar (PDA; Difco, Detroit, MI, USA) and dichloran rose bengal chloramphenicol agar (DRBC; Difco, USA) were used. Using 5 g of soil, serial dilutions were conducted, and 0.1 mL of 1/100 and 1/1000 dilutions were spread on agar plates. Two PDA and two DRBC plates were prepared for each dilution and incubated at 25 °C for 2–7 days. Fungal colonies were sub-cultured in PDA medium to obtain pure culture and incubated at 25 °C. They were grouped based on the growth morphologies such as growth rate and hyphal morphology. One to three isolates of each group were further identified molecularly.

### 2.2. Molecular Experiment and Phylogenetic Analysis for Identification

Genomic DNA was extracted using a modified cetyltrimethylammonium bromide (CTAB) method [35]. PCR amplification was conducted using AccuPower PCR PreMix kit (Bioneer, Daejeon, South Korea). The ITS region was amplified for all fungal isolates using ITS1F and ITS4 primers [36]. For *Penicillium*, the beta tubulin (*benA*) gene was also amplified using Bt2a and Bt2b primers [37], and translation elongation factor 1-alpha (*tef1α*) gene for *Trichoderma* was amplified using EF1-728F [38] and TEF1rev [39] primers. We followed previous studies for PCR conditions and purification procedures [25,40,41]. Sequencing was conducted at Macrogen (Seoul, South Korea) using an Applied Biosystems 3730 genetic analyzer (Life Technologies, Gaithersburg, MD, USA).

Sequences were proofread and edited using MEGA v. 5.2 [42] and aligned with reference sequences using MAFFT v. 7 [43]. Phylogenetic analysis was performed using the neighbor-joining method with a Kimura-2 parameter model and 1000 bootstrap replications. Sequences generated from this study were deposited at GenBank under accession number MK789182-MK789209 for ITS, MK800126-MK800141 for *benA*, and MK800142-MK800145 for *tef1α*.

The community structures of microfungi in PM fairy rings were analyzed using a constrained analysis of principal coordinates (CAP) analysis based on binary Jaccard dissimilarities using the phyloseq package [44] in R [45]. CAP analysis was used to compare the presence or absence of microfungi species between sampling locations.

### 2.3. Effect of Fungal Metabolites on PM Growth

Metabolites of microfungi were extracted to investigate their effects on PM growth. We chose a representative isolate randomly from each fungal species and cultured it for 14 days on five 90-mm PDA plates. The contents of all five plates were transferred to a beaker containing 300 mL of 80% methanol and chopped by spatula. After one day of incubation, the solution was filtered through 150 mm Whatman filter paper (Advantec, Tokyo, Japan). The filtered solution was concentrated using an EYELA rotary vacuum evaporator N-N series (Tokyo Rikakikai, Japan) to a final volume of 5 mL. In a blank Petri dish, 50 µL of the concentration were inoculated on a sterilized paper disc (8 mm; Advantec, Japan) and methanol was evaporated by air drying for 12 hours. The growth experiment was conducted on ‘Tricholoma matsutake media’ (TMM) (glucose 20 g/L, yeast extract 1.5 g/L, soytone 1.5 g/L, and agar 20 g/L) [46]. PM isolate (KMRB 12100405) was provided by Korea Mushroom Resource Bank (Seoul, Republic of Korea). PM isolate was cultured in potato dextrose broth (PDB; Difco, USA) at 25 °C for 6 months and homogenized in 30 mL of sterilized distilled water. Subsequently, 20 µL of homogenized PM isolate were inoculated on the center of a 60-mm Petri dish containing TMM, and the paper disc inoculated with fungal extract was placed 15 mm away from the PM inoculant. The experiment was conducted to five replicates and incubated at 25 °C for 4 weeks (28 days). The growth area of PM isolate was measured three times using ImageJ2 [47] with SIOX plugin [48] and averaged. The PM growth area was compared between control plates (PM isolate grown without fungal metabolites) and treatment plates (PM isolate grown with fungal metabolite). Differences were tested using pairwise Wilcoxon rank-sum tests corrected by false discovery rate according to Benjamini and Hochberg [49].

## 3. Results

### 3.1. Species Identification and Composition

**Figure 1:**
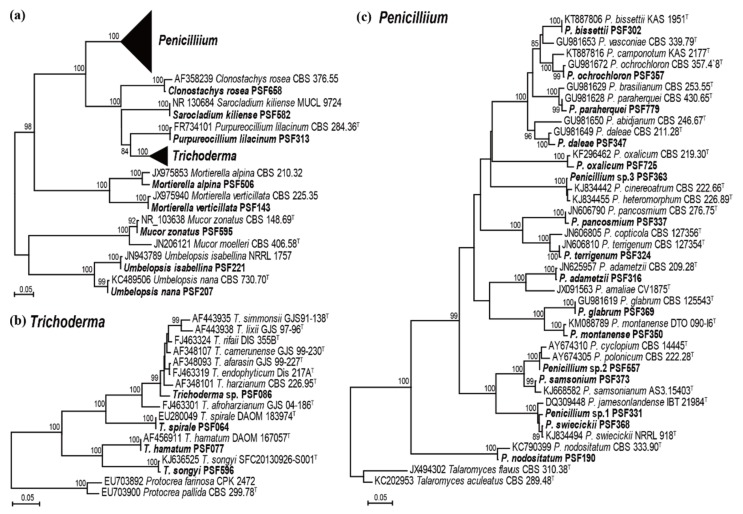
Diversity of microfungi isolated from soil within pine mushroom (PM) fairy rings. Phylogenetic tree based on (**a**) ITS sequences for all species, (**b**) tef1α sequences for *Trichoderma*, and (**c**) *benA* sequences for *Penicillium* species. Phylogenetic trees were constructed based on the neighbor-joining method with the Kimura-2-parameter model and 1000 bootstrap replications. Bootstrap values (>70) are presented on the branch. “T” represents ex-type, and sequences generated in this study are in bold.

A total of 184 fungal isolates were obtained from soil within PM fairy rings at four sampling locations. Based on the growth morphologies and ITS sequences, microfungi were initially grouped into 28 taxa. Isolates were identified to species level by phylogenetic analysis using ITS region (Figure 1a), except for *Penicillium* and *Trichoderma,* which were identified with further partial sequences of *benA* gene (Figure 1b) and *tef1α* gene (Figure 1c), respectively. For these two genera, phylogenetic analyses using the ITS region showed low resolution, which was not adequate for identification to the species level. Twenty-four species were assigned to species level, whereas four species, three *Penicillium* and one *Trichoderma*, remained unidentified because of the unclear phylogenetic relationships. Twenty-eight species spanned two phyla, four classes, five orders, seven families, and eight genera.

**Figure 2:**
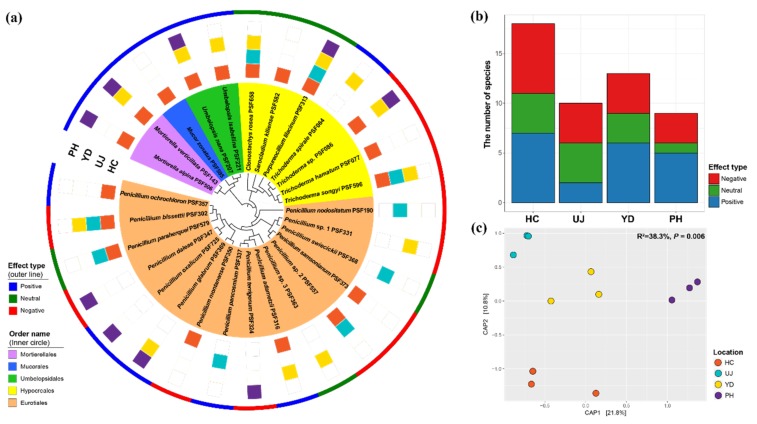
Patterns of occurrence and effect type for microfungal species isolated from soil within PM fairy rings. (**a**) Phylogenetic tree with information for isolation location and effect type on PM growth. The phylogenetic tree was constructed based on ITS sequences from the strain used for metabolite experiments. Colors in the box indicate the presence of the species in each location. Colors in the outer line indicate effect type on PM growth. (**b**) Number of species isolated from four locations. (**c**) Constrained analysis of principal coordinates (CAP) plots for community structure based on binary Jaccard dissimilarity. CAP model constrained by sampling locations (*p* = 0.006; 38.3% explanatory power). HC: Hongcheon site, UJ: Uljin site, YD: Yeongdeok site, PH: Pohang site.

Eurotiales and Hypocreales belonging to Ascomycota had the largest species number, with 16 and 7 species, respectively (Figure 2a). *Penicillium* showed high species richness (16 species), followed by *Trichoderma* (4 species). For the other Ascomycetes species, *Clonostachys rosea*, *Sarocladium kiliense*, and *Purpureocillium lilacinum* were also isolated. In Zygomycota, *Mucor zonatus*, two *Mortierella* species, and two *Umbelopsis* species were isolated. Among the 28 fungal species, only *Purpureocillium lilacinum* was isolated from all locations (Figure 2a). Six species (*Clonostachys rosea, Mortierella verticillata, Penicillium bissettii, Trichoderma songyi, Trichoderma* sp., and *Umbelopsis nana*) were isolated from three locations. Fifteen species were isolated only from a single location. *Penicillium bissettii* was most frequently detected (7 fairy rings), followed by *Purpureocillium lilacinum* (6 fairy rings) and *Mortierella verticillata* (5 fairy rings) (Table S1). Among the four sampling locations, Hongcheon had the highest number of species (Figure 2b).

Compositions of microfungi in PM fairy rings were compared using CAP analysis, which showed different distributional patterns according to geography (Figure 2c). Fungal communities were significantly different between the sampling locations (*p* = 0.006; 38.3% explanatory power) based on binary Jaccard dissimilarity.

### 3.2. Effect of Fungal Metabolite on Mycelial Growth of PM

**Figure 3:**
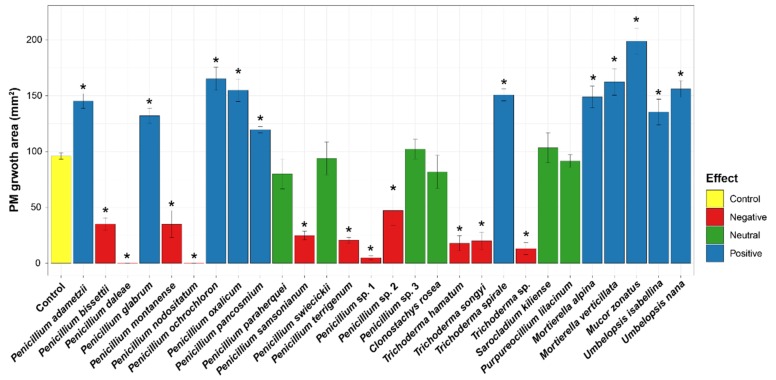
Effect of microfungal metabolite on PM growth. Average radial growth area (mm^2^) of PM grown with microfungal metabolites was measured. The strain numbers used for the experiment are presented in Figure 2a. The growth of PM cultured with metabolite was compared to that of control plates using pairwise Wilcoxon tests with multiple test corrections according to the false discovery rate of Benjamini and Hochberg. An asterisk indicates a significant difference (*: *p* < 0.05).

A representative strain from each species was chosen for testing the effect of fungal metabolite on PM growth (Figure 3). Among 28 species, 11 species showed a significant positive effect on PM growth, while 11 species had a significantly negative effect (Figure 3). All species in Zygomycota (*Mortierella alpina*, *Mortierella verticillata*, *Mucor zonatus*, *Umbelopsis isabellina*, and *Umbelopsis nana*), four *Penicillium* species, and *Trichoderma spirale* promoted PM growth; the treatment increased PM growth area by 124–207% compared with the control plates. *Mucor zonatus* had the highest growth promoting effect (207%), followed by *Penicillium ochrochloron* (172%) and *Mortierella verticillata* (169%). In contrast, eight *Penicillium* species (*P. bissettii, P. daleae, P. montanense, P. nodositatum, P. samsonianum, P. terrigenum, Penicillium* sp.1, and *Penicillium* sp.2) and three *Trichoderma* species suppressed PM growth; the treatment decreased PM growth by 0–49% compared with the control plates. *Penicillium daleae* and *P. nodositatum* had the most negative effects on PM growth, as PM isolates did not grow on the plates treated with the metabolites from these *Penicillium* species.

A phylogenetic signal based on the effects of fungal metabolites on PM growth (‘effect type’) was detected in Zygomycota, with all species having a positive effect on PM growth (Figure 2a). In Ascomycota, however, the distribution of effect types was different depending on the species even within the same genus. Within the sampling locations, the number of species was not statistically different between effect types (*p* = 0.913), although Uljin and Pohang had a relatively low number of positive and neutral effect types, respectively (Figure 2b). When the communities were separated by effect type, the community structures of negative types were significantly different between locations (*p* = 0.001; 48.3% explanatory power), while positive and neutral fungal communities were not significantly different (*p* = 0.167 and 0.602, respectively) (Figure S1).

## 4. Discussion

Many microfungi isolated from rhizosphere soil belong to *Aspergillus*, *Penicillium*, and *Trichoderma* [15]. Species of *Aspergillus, Mortierella, Mucor*, and *Penicillium* were also reported from PM fairy ring soil [23,24,26]. Species within the genus often have similar growth profiles, which leads to misidentification [50,51]. In previous studies, these were often designated without a species epithet. The recent development of molecular approaches based on nucleotide sequences resulted in better resolution to distinguish species with a similar growth morphology [52,53,54]. Although the ITS sequence is used as the fungal barcode [33], *benA* and *tef1α* are more efficiently used for the identification of *Penicillium* and *Trichoderma*, respectively [49,50]. The use of suitable markers for a particular group seems to increase species diversity and identification accuracy.

In this study, we identified many microfungi to species level with appropriate molecular markers. A higher number of *Penicillium* species (16 species) was found compared with a previous report [26]. *Penicillium bissettii* was the most commonly isolated species from three locations, followed by *Penicillium adametzii, Penicillium glabrum, Penicillium paraherquei*, and *Penicillium* sp. 2 from two locations (Figure 2a). Among these, *Penicillium glabrum* was the only species matched with a previous report on fungal diversity in PM fairy ring soil [26]. You et al. [9] isolated 10 *Penicillium* species from pine roots in PM fairy rings and two species (*P. montanense* and *P. swiecickii*) matched with our results. Among the four *Trichoderma* species isolated from PM fairy rings, *T. songyi* and *Trichoderma* sp. were frequently isolated. Three *Trichoderma* species (*T. hamatum, T. songyi,* and *T. spirale*) were previously detected from pine roots within PM fairy rings [33]. *Purpureocillium lilacinum* found in all sampling locations is known as nematophagous fungus and used as a biocontrol agent against a root-knot nematode [55,56]. Given that the abundance of nematodes is higher in fungal-mycelia-dominant environments [57], *Purpureocillium lilacinum* may be recruited to PM fairy rings for hunting nematodes.

*Mortierella* and *Umbelopsis* are frequently reported genera associated with PM fairy ring soil, roots, and fruiting bodies [9,11,23,24]. Similarly, either *Mortierella* or *Umbelopsis* species were detected at all locations in this study (Figure 2a). While our result showed that genera found in PM fairy rings largely matched those in previous studies, only a few of them were consistent at the species level. This seems to be the result of different approaches in identification, since previous studies mostly implemented morphological approaches or BLAST analysis only. Such an approach may have precluded the accurate identification of isolates to the species level.

Metabolites extracted from microfungi had various effects on PM growth (Figure 3). Metabolites from 11 species promoted PM mycelial growth (Figure 3). The phylogenetic distribution of effect types was different between Zygomycota and Ascomycota (Figure 2a). In Zygomycota, all species isolated from PM fairy ring soil showed a high promoting effect on PM growth. Ogawa and Kawai [32] also reported that a metabolite from *Mortierella* species can promote PM growth. These results suggest that the growth-promoting effect may be phylogenetically conserved within the zygomycete species. In Ascomycota, however, species with positive and negative effects were evenly distributed on broad taxonomic ranges (Figure 2a). Given the fact that congeneric species of Ascomycota showed the opposite effect on PM growth, the species trait of positive effect was not conserved phylogenetically. Among the Ascomycetes species, *Penicillium adametzii, Penicillium ochrochloron, Penicillium oxalicum, Penicillium pancosmium*, and *Trichoderma spirale* showed a positive effect (Figure 3). In our previous study, PM-growth-promoting bacteria promoted the growth of *Penicillium oxalicum* and *Umbelopsis nana*; thus, these species were speculated to have a positive effect on PM, as PM-growth-promoting bacteria may have the additional effect of increasing PM growth indirectly by promoting the growth of these fungi [58]. This hypothesis was confirmed by this study. The results of this study are also in line with our previous study showing that *Trichoderma spirale* promoted PM growth [54]. The exact mechanism of promoting PM growth by these fungi is uncertain, yet it is suspected that additional nutrition secreted by microfungi or fungal metabolites may possibly stimulate hyphal extension. A similar effect from bacterial metabolite was reported from MHB *Streptomyces* sp. AcH 505, which produces auxofuran associated in promoting the growth of ectomycorrhizal fungus, *Amanita muscaria* [59]. Because the effect on ectomycorrhizal colonization is often related to a hyphal-growth-promotion effect [60], several microfungi exhibiting a positive effect on PM growth in this study may be considered as mycorrhiza helper fungus for PM, as previously proposed for *Arthrinium phaeospermum* for the growth of *Tuber borchii* [61].

Although our results are generally in line with previous studies, it should be noted that our results pertain to the interaction between a particular strain of PM and a microfugal isolate. Because of the intraspecific variation in fungal activity, the effect of microfungi on PM growth can differ depending on the strain. For example, *Trichoderma songyi* PF052 isolated from pine root showed a positive effect on PM growth [33], while *T. songyi* PSF596 in this study significantly suppressed PM growth when we used the same PM isolate as in the previous study [33] (Figure 3). In addition, the mode of interaction (e.g. growth promotion or suppression) can be changed depending on the strain, either PM or microfungi, due to the evolutionary history between two species that may have adapted to each other. In the case of bacteria, the effect on the growth of ectomycorrhizal fungi, *Laccaria bicolor* and *Laccaria parva*, was different depending on strain pairs [62,63]. Interactions between PM and microfungi need to be verified in further studies.

Communities of PM-associated microfungi differed by geographical location (Figure 2c). Such differences were also observed in a previous metabarcoding study with fungal communities in soil from PM fairy rings [28]. Given that the microfungi in PM fairy rings are recruited from local species pools, different environmental conditions may create different species pools in local communities. The community structure differed by effect type of microfungi (Figure S1). The fungal communities of negative types were relatively local specific, while the fungal compositions of positive or neutral species were largely shared between locations. Such an observation suggests that the positive and neutral microfungi have a more intimate relationship with PM than does the negative type.

## 5. Conclusions

We identified 28 species of microfungi from PM fairy rings. The accurate identification of microfungi based on taxa-specific markers is crucial for understanding the complexity of microfungi–EMF interaction. We found 11 microfungi promoting the mycelial growth (two *Mortierella* species, *Mucor zonatus*, five *Penicillium* species, *Trichoderma spirale*, and two *Umbelopsis* species). Although metabolite effects on PM growth are variable between species within the genus, microfungi with a positive effect have the potential for being used as mycorrhiza helper fungus for PM. Using these helper fungi, we may be able to influence forest nursery and advance the artificial cultivation of EMF including PM.

## Supplementary Materials

Supplementary materials can be found at https://www.mdpi.com/2076-2607/7/6/169/s1: Figure S1: Constrained analysis of principal coordinates (CAP) plots for community structures separated by effect type; Table S1: Number of PM fairy rings harboring the microfungal species.

## References

[1] Landeweert R., Hoffland E., Finlay R.D., Kuyper T.W., van Breemen N. (2001). Linking plants to rocks: Ectomycorrhizal fungi mobilize nutrients from minerals. Trends Ecol. Evol..

[2] Bonfante P., Genre A. (2010). Mechanisms underlying beneficial plant–fungus interactions in mycorrhizal symbiosis. Nat. Commun..

[3] Bonfante P., Hock B. (2001). At the interface between mycorrhizal fungi and plants: The structural organization of cell wall, plasma membrane and cytoskeleton. The Mycota IX: Fungal Associations.

[4] Garbaye J. (1994). Helper bacteria: A new dimension to the mycorrhizal symbiosis. New Phytol..

[5] Frey-Klett P., Garbaye J., Tarkka M. (2007). The mycorrhiza helper bacteria revisited. New Phytol..

[6] Kiers E.T., Duhamel M., Beesetty Y., Mensah J.A., Franken O., Verbruggen E., Fellbaum C.R., Kowalchuk G.A., Hart M.M., Bago A. (2011). Reciprocal rewards stabilize cooperation in the mycorrhizal symbiosis. Science.

[7] Wyatt G.A., Kiers E.T., Gardner A., West S.A. (2014). A biological market analysis of the plant-mycorrhizal symbiosis. Evolution.

[8] Tedersoo L., Pärtel K., Jairus T., Gates G., Põldmaa K., Tamm H. (2009). Ascomycetes associated with ectomycorrhizas: Molecular diversity and ecology with particular reference to the Helotiales. Environ. Microbiol..

[9] You Y.-H., Yoon H.-J., Woo J.-R., Rim S.-O., Lee J.-H., Kong W.-S., Kim J.-G. (2011). Diversity of endophytic fungi isolated from the rootlet of *Pinus densiflora* colonized by *Tricholoma matsutake*. Korean J. Mycol..

[10] Kluber L.A., Smith J.E., Myrold D.D. (2011). Distinctive fungal and bacterial communities are associated with mats formed by ectomycorrhizal fungi. Soil Biol. Biochem..

[11] Li Q., Chen Ch., Penttinen P., Xiong Ch., Zheng L., Huang W. (2016). Microbial diversity associated with *Tricholoma matsutake* fruiting bodies. Microbiology.

[12] Pacioni G., Leonardi M., Aimola P., Ragnelli A.M., Rubini A., Paolocci F. (2007). Isolation and characterization of some mycelia inhabiting *Tuber ascomata*. Mycol. Res..

[13] Baar J., Stanton N.L. (2000). Ectomycorrhizal fungi challenged by saprotrophic basidiomycetes and soil microfungi under different ammonium regimes in vitro. Mycol. Res..

[14] Leake J.R., Donnelly D.P., Boddy L., van der Heijden M.G.A., Sanders I.R. (2003). Interactions between ecto-mycorrhizal and saprotrophic fungi. Mycorrhizal ecology.

[15] Summerbell R.C. (2005). From Lamarckian fertilizers to fungal castles: Recapturing the pre-1985 literature on endophytic and saprotrophic fungi associated with ectomycorrhizal root systems. Stud. Mycol..

[16] Mucha J., Zadworny M., Werner A., Napierala-Filipiak A., Lakomy P. (2008). Antagonistic activity of the ectomycorrhizal fungus *Suillus bovinus* challenged by saprotrophic fungi from different soils. Nova Hedwig..

[17] Kope H.H., Fortin J.A. (1989). Inhibition of phytopathogenic fungi in vitro by cell free culture media of ectomycorrhizal fungi. New Phytol..

[18] Whipps J.M. (2004). Prospects and limitations for mycorrhizas in biocontrol of root pathogens. Can. J. Bot..

[19] Malyshkin P.E. (1955). Stimulation of tree growth by microorganisms. Mycotrophy in Plants.

[20] Voznyakovskaya Y.M., Ryzhkova A. (1955). Microflora accompanying mycorrhizas. Mycotrophy of Woody Plants.

[21] Yun W., Hall I.R., Evans L.A. (1997). Ectomycorrhizal fungi with edible fruiting bodies 1. *Tricholoma matsutake* and Related Fungi. Econ. Bot..

[22] Yamanaka T., Ota Y., Konno M., Kawai M., Ohta A., Neda H., Terashima Y., Yamada A. (2014). The host ranges of conifer-associated *Tricholoma matsutake*, Fagaceae-associated *T. bakamatsutake* and *T. fulvocastaneum* are wider in vitro than in nature. Mycologia.

[23] Kataoka R., Siddiqui Z.A., Kikuchi J., Ando M., Sriwati R., Nozaki A., Futai K. (2012). Detecting nonculturable bacteria in the active mycorrhizal zone of the pine mushroom *Tricholoma matsutake*. J. Microbiol..

[24] Ohara H., Hamada M. (1967). Disappearance of bacteria from the zone of active mycorrhizas in *Tricholoma matsutake* (S. Ito et Imai) Singer. Nature.

[25] Park M.S., Oh S.-Y., Cho H.J., Fong J.J., Cheon W.-J., Lim Y.W. (2014). *Trichoderma songyi* sp. nov., a new species associated with the pine mushroom (*Tricholoma matsutake*). Antonie Van Leeuwenhoek.

[26] Song H.-S., Min K.-H. (1991). Microfungal flora of *Tricholoma matsutake* producing and nonproducing sites in the forest of *Pinus densiflora*. Korean J. Mycol..

[27] Kim M., Yoon H.J., You Y.H., Kim Y.E., Woo J.R., Seo Y.G., Lee G.M., Kim Y.J., Kong W.S., Kim J.G. (2013). Metagenomic analysis of fungal communities inhabiting the fairy ring zone of *Tricholoma matsutake*. J. Microbiol. Biotechnol..

[28] Oh S.-Y., Fong J.J., Park M.S., Lim Y.W. (2016). Distinctive feature of microbial communities and bacterial functional profiles in *Tricholoma matsutake* dominant soil. PLoS ONE.

[29] Vaario L.-M., Fritze H., Spetz P., Heinonsalo J., Hanajík P., Pennanen T. (2011). *Tricholoma matsutake* dominates diverse microbial communities in different forest soils. Appl. Environ. Microbiol..

[30] Oh S.-Y., Lim Y.W. (2018). Effect of fairy ring bacteria on the growth of *Tricholoma matsutake* in vitro culture. Mycorrhiza.

[31] Oh S.-Y., Lim Y.W. (2018). Root-associated bacteria influencing mycelial growth of *Tricholoma matsutake* (pine mushroom). J. Microbiol..

[32] Ogawa M., Kawai M. (1976). Studies on the artificial reproduction of *Tricholoma matsutake* (S. Ito et Imai) Sing. III. Effects of growth promotion of natural products on the vegetative growth of *T. matsutake*. Trans. Mycol. Soc. Jpn..

[33] Oh S.-Y., Park M.S., Cho H.J., Lim Y.W. (2018). Diversity and effect of *Trichoderma* isolated from the roots of *Pinus densiflora* within the fairy ring of pine mushroom (*Tricholoma matsutake*). PLoS ONE.

[34] Schoch C.L., Seifert K.A., Huhndorf S., Robert V., Spouge J.L., Levesque C.A., Chen W., Consortium F.B. (2012). Nuclear ribosomal internal transcribed spacer (ITS) region as a universal DNA barcode marker for Fungi. Proc. Natl. Acad. Sci. USA.

[35] Rogers S.O., Bendich A.J., Gelvin S.B., Schilperoort R.A. (1994). Extraction of total cellular DNA from plants, algae and fungi. Plant Molecular Biology Manual.

[36] White T.J., Bruns T., Lee S., Taylor J.L. (1990). Amplification and direct sequencing of fungal ribosomal RNA genes for phylogenetics. PCR protocols: A guide to methods and applications.

[37] Glass N.L., Donaldson G.C. (1995). Development of primer sets designed for use with the PCR to amplify conserved genes from filamentous ascomycetes. Appl. Environ. Microbiol..

[38] Carbone I., Kohn L.M. (1999). A Method for designing primer sets for speciation studies in filamentous Ascomycetes. Mycologia.

[39] Samuels G.J., Dodd S.L., Gams W., Castlebury L.A., Petrini O. (2002). *Trichoderma* species associated with the green mold epidemic of commercially grown *Agaricus bisporus*. Mycologia.

[40] Park M.S., Fong J.J., Oh S.-Y., Houbraken J., Sohn J.H., Hong S.-B., Lim Y.W. (2015). *Penicillium jejuense* sp. nov., isolated from the marine environments of Jeju Island, Korea. Mycologia.

[41] Park M.S., Fong J.J., Lee H., Oh S.-Y., Jung P.E., Min Y.J., Seok S.J., Lim Y.W. (2013). Delimitation of *Russula* Subgenus *Amoenula* in Korea Using Three Molecular Markers. Mycobiology.

[42] Tamura K., Peterson D., Peterson N., Stecher G., Nei M., Kumar S. (2011). MEGA5: Molecular evolutionary genetics analysis using maximum likelihood, evolutionary distance, and maximum parsimony methods. Mol. Biol. Evol..

[43] Katoh K., Standley D.M. (2013). MAFFT multiple sequence alignment software version 7: Improvements in performance and usability. Mol. Biol. Evol..

[44] McMurdie P.J., Holmes S. (2013). Phyloseq: An R package for reproducible interactive analysis and gaphics of microbiome census data. PLoS ONE.

[45] R Core Team R: A language and environment for statistical computing. https://www.R-project.org/.

[46] Kim I.-Y., Jung G.-R., Han S.-K., Cha J.-Y., Sung J.-M. (2005). Favorable condition for mycelial growth of *Tricholoma matsutake*. Korean J. Mycol..

[47] Rueden C.T., Schindelin J., Hiner M.C., DeZonia B.E., Walter A.E., Arena E.T., Eliceiri K.W. (2017). ImageJ2: ImageJ for the next generation of scientific image data. BMC Bioinform..

[48] Wang F. (2017). SIOX plugin in ImageJ: Area measurement made easy. UV4Plants Bull..

[49] Benjamini Y., Hochberg Y. (1995). Controlling the False Discovery Rate: A Practical and Powerful Approach to Multiple Testing. J. R. Stat. Soc. Ser. B Methodol..

[50] Druzhinina I.S., Kopchinskiy A.G., Kubicek C.P. (2006). The first 100 *Trichoderma* species characterized by molecular data. Mycoscience.

[51] Visagie C.M., Houbraken J., Frisvad J.C., Hong S.-B., Klaassen C.H.W., Perrone G., Seifert K.A., Varga J., Yaguchi T., Samson R.A. (2014). Identification and nomenclature of the genus *Penicillium*. Stud. Mycol..

[52] Chaverri P., Castlebury L.A., Samuels G.J., Geiser D.M. (2003). Multilocus phylogenetic structure within the *Trichoderma harzianum/Hypocrea lixii* complex. Mol. Phylogenet. Evol..

[53] Perrone G., Stea G., Epifani F., Varga J., Frisvad J.C., Samson R.A. (2011). *Aspergillus niger* contains the cryptic phylogenetic species *A. awamori*. Fungal Biol..

[54] Taylor J.W., Jacobson D.J., Kroken S., Kasuga T., Geiser D.M., Hibbett D.S., Fisher M.C. (2000). Phylogenetic species recognition and species concepts in fungi. Fungal Genet. Biol..

[55] Anastasiadis I.A., Giannakou I.O., Prophetou-Athanasiadou D.A., Gowen S.R. (2008). The combined effect of the application of a biocontrol agent *Paecilomyces lilacinus*, with various practices for the control of root-knot nematodes. Crop Prot..

[56] Liu J., Sun J., Qiu J., Liu X., Xiang M. (2014). Integrated management of root-knot nematodes on tomato in glasshouse production using nematicides and a biocontrol agent, and their effect on soil microbial communities. Nematology.

[57] Cromack K., Fichter B.L., Moldenke A.M., Entry J.A., Ingham E.R. (1988). Interactions between soil animals and ectomycorrhizal fungal mats. Agric. Ecosyst. Environ..

[58] Oh S.-Y., Kim M., Eimes J.A., Lim Y.W. (2018). Effect of fruiting body bacteria on the growth of *Tricholoma matsutake* and its related molds. PLoS ONE.

[59] Riedlinger J., Schrey S.D., Tarkka M.T., Hampp R., Kapur M., Fiedler H.-P. (2006). Auxofuran, a novel metabolite that stimulates the growth of fly agaric, is produced by the mycorrhiza helper bacterium *Streptomyces* strain AcH 505. Appl. Environ. Microbiol.

[60] Brulé C., Frey-Klett P., Pierrat J.C., Courrier S., Gérard F., Lemoine M.C., Rousselet J.L., Sommer G., Garbaye J. (2001). Survival in the soil of the ectomycorrhizal fungus *Laccaria bicolor* and the effects of a mycorrhiza helper *Pseudomonas fluorescens*. Soil Biol. Biochem..

[61] Sabella E., Nutricati E., Aprile A., Miceli A., Sorce C., Lorenzi R., de Bellis L. (2015). *Arthrinium phaeospermum* isolated from *Tuber borchii* ascomata: The first evidence for a “Mycorrhization Helper Fungus”?. Mycol. Prog..

[62] Labbé J.L., Weston D.J., Dunkirk N., Pelletier D.A., Tuskan G.A. (2014). Newly identified helper bacteria stimulate ectomycorrhizal formation in *Populus*. Front. Plant. Sci..

[63] Obase K. (2019). Extending the hyphal area of the ectomycorrhizal fungus *Laccaria parva* co-cultured with ectomycorrhizosphere bacteria on nutrient agar plate. Mycoscience.

