# Supplementary material for: The Influence of Microfungi on the Mycelial Growth of Ectomycorrhizal Fungus Tricholoma matsutake
Source: Microorganisms. 2019 Jun 7;7(6):169. doi: 10.3390/microorganisms7060169 (PMC6617177; doi:10.3390/microorganisms7060169)
Supplement: Supplementary file 1 [file microorganisms-07-00169-s001.zip › Table S1.pdf]

Table S1. The number of PM fairy rings harboring the microfungal species.

Species name	Effect[1)]	Location[2)]	The number of fairy ring				
			Total	HC	UJ	YD	PH
Penicillium adametzii	Positive	2	2	1	0	1	0
Penicillium bissettii	Negative	3	7	2	3	2	0
Penicillium daleae	Negative	1	1	0	0	0	1
Penicillium glabrum	Positive	2	4	0	0	1	3
Penicillium montanense	Negative	1	3	3	0	0	0
Penicillium nodositatum	Negative	1	1	0	1	0	0
Penicillium ochrochloron	Positive	1	1	1	0	0	0
Penicillium oxalicum	Positive	1	1	0	0	0	1
Penicillium pancosmium	Positive	1	1	0	1	0	0
Penicillium paraherquei	Neutral	2	2	1	1	0	0
Penicillium samsonianum	Negative	1	2	2	0	0	0
Penicillium swiecickii	Negative	1	1	0	1	0	0
Penicillium terrigenum	Negative	1	2	0	0	0	2
Penicillium sp.1	Neutral	1	2	0	0	2	0
Penicillium sp.2	Neutral	2	2	1	1	0	0
Penicillium sp.3	Negative	1	1	0	0	1	0
Clonostachys rosea	Neutral	3	3	1	1	1	0
Trichoderma hamatum	Negative	1	1	1	0	0	0
Trichoderma songyi	Negative	3	3	1	1	1	0
Trichoderma spirale	Negative	1	1	0	0	1	0
Trichoderma sp.	Positive	3	4	1	0	1	2
Sarocladium kiliense	Neutral	1	2	2	0	0	0
Purpureocillium lilacinum	Neutral	4	6	1	2	1	2
Mortierella alpina	Positive	3	3	1	1	0	1
Mortierella verticillata	Positive	3	5	1	0	1	3
Mucor zonatus	Positive	1	1	1	0	0	0
Umbelopsis isabellina	Positive	2	2	1	0	1	0
Umbelopsis nana	Positive	3	4	2	0	1	1

1) The effect type of microfungal species on the PM growth

2) The number of locations harboring the microfungal species
