# Supplementary material for: The Influence of Microfungi on the Mycelial Growth of Ectomycorrhizal Fungus Tricholoma matsutake
Source: Microorganisms. 2019 Jun 7;7(6):169. doi: 10.3390/microorganisms7060169 (PMC6617177; doi:10.3390/microorganisms7060169)

Supplementary Figure 1. Constrained Analysis of Principal coordinates (CAP) plots for community structures separated by effect type. The communities were comprised by the microfungi with (A) positive effect, (B) neutral effect, and (C) negative effect. CAP model constrained by locations was tested based on binary Jaccard dissimilarity HC: Hongcheon site, UJ: Uljin site, YD: Yeongdeok site, PH: Pohang site.

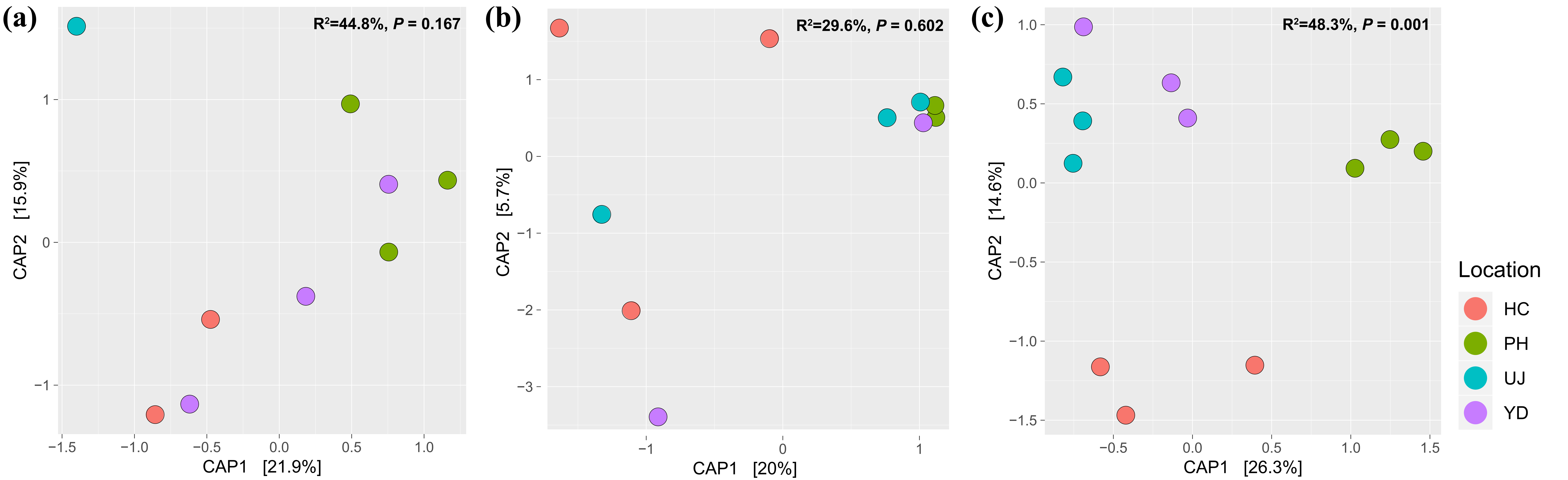

Supplement: Supplementary file 1 [file microorganisms-07-00169-s001.zip › Figure S1.pdf]
